# The vertical pattern of microwave radiation around BTS (Base Transceiver Station) antennae in Hashtgerd township

**DOI:** 10.1186/2052-336X-11-40

**Published:** 2013-12-20

**Authors:** Simin Nasseri, Mohammadreza Monazzam, Meisam Beheshti, Sajad Zare, Amirhosein Mahvi

**Affiliations:** 1 Department of Environmental Health Engineering, School of Public Health, Tehran University of Medical Sciences, Tehran, Iran; 2 Center for Water Quality Research, Institute for Environmental Research, Tehran University of Medical Sciences, Tehran, Iran; 3 Department of Occupational Hygiene, School of Public Health, Tehran University of Medical Sciences, Tehran, Iran; 4 Center for Air Quality Research, Institute for Environmental Research, Tehran University of Medical Sciences, Tehran, Iran

**Keywords:** BTS antennae, Potential density, Electromagnetic waves, Microwave vertical measuring

## Abstract

New environmental pollutants interfere with the environment and human life along with technology development. One of these pollutants is electromagnetic field. This study determines the vertical microwave radiation pattern of different types of Base Transceiver Station (BTS) antennae in the Hashtgerd city as the capital of Savojbolagh County, Alborz Province of Iran. The basic data including the geographical location of the BTS antennae in the city, brand, operator type, installation and its height was collected from radio communication office, and then the measurements were carried out according to IEEE STD 95. 1 by the SPECTRAN 4060. The statistical analyses were carried out by SPSS16 using Kolmogorov Smirnov test and multiple regression method. Results indicated that in both operators of Irancell and Hamrah-e-Aval (First Operator), the power density rose with an increase in measurement height or decrease in the vertical distance of broadcaster antenna. With mix model test, a significant statistical relationship was observed between measurement height and the average power density in both types of the operators. With increasing measuring height, power density increased in both operators. The study showed installing antennae in a crowded area needs more care because of higher radiation emission. More rigid surfaces and mobile users are two important factors in crowded area that can increase wave density and hence raise public microwave exposure.

## Introduction

Nowadays living in the globe, indeed, is like swimming in the sea of natural electromagnetic fields. During the past century, this natural environment has changed a lot due to the presence of artificial electromagnetic fields. Using the new technology by developing countries followed by developed countries has brought higher wave exposure risk for public [1,2]. Electromagnetic waves in different industries, communications, science, medicine and home appliances is important [3]. Many people are increasingly affected by the artificial energy fields in mobile phones, in which the technology uses microwaves with wide range of frequency spectrum usually starts from 900 MHz [4]. There is also a basic difference between facing mobile waves and facing waves of BTS antennae, which has not gained much attention. Because receiving mobile waves is non-permanent and usually last for a short time, but exposure to the BTS antennae can be for 24 hours and it can be going on for years [5]. Since a large number of BTS antennae have been installed in the cities, people are worried about exposure to these waves. For protection against thermal effects of these exposures, a maximum rate of exposure to electromagnetic field based on international organizations such as ICNIRP have been introduced [6]. Hutter et al. in 2006 studied the effective rate of the magnetic fields of 10 BTS antennae in Austria (5 BTS in cities and 5BTS in villages) and found out that the rates were varied between 0.0002-4.1 mw/m^2^[7].

A study by Safari et al. in 1997 determined the effective rate of electromagnetic field and X- ray levels in different parts of Mehrabad Airport (tower and approach, transmitter, radar, and navigation). They found that the amount of electric field strength, Magnetic field strength and X- Ray exposure respectively were: Tower and approach (10 v/m, 0.0001 A/m, 0.0001 mR/h), transmitter (10 v/m, 0.7 A/m, 0.01 mR/h), radar (5.77 v/m, 0.02 A/m, 5 mR/h) and navigation (10 v/m, 0.07 A/m, 0.1 mR/h) [3].

Many epidemiological studies have shown health effects such as reproductive problems from exposure to electromagnetic fields and radiation [8]. A study by Ferreri et al. in 2006 indicated that intra-cortical excitability curve was significantly modified during real exposure, with short intra-cortical inhibition being reduced and facilitation enhanced in the acutely exposed brain hemisphere [9]. Due to the tall buildings in big cities and doubts on the quality of density of radiation microwaves by increasing measuring height around the BTS antennae in Hashtgerd city, this study was carried out to answer this question that how the field pattern in the distance of 0-16.5 m around the BTS antennae was changed by increasing height.

## Materials and methods

In the present study, which is a descriptive-analytical study, BTS antenna with 900-1800 MHz frequency are assumed as the main sources of electromagnetic field in the city of Hashtgerd. The required data such as number and location of BTS antennae were gathered, and organized by their operator type, brand and technical characterizations, obtained from Radio Communications Office, Environmental Protection Agency, Telecommunications Organization and other related organizations [10]. The gathered data was completed by double-checking and field visits. Measuring of the variables were carried out by the calibrated Spectran-4060 and the standard method of IEEE StdC95.1. Measurements in different heights were carried out using a crane installed in 15 m distance from the tower.

It is worth to add that due to the size required to create a far-field range for this kind of antennas, near-field techniques was used, which allow the measurement of the field on a surface closer to the antenna. The effective field around the antenna (R_eff_) was estimated from the below equation:

(1) $$R_{e\mathrm{ff}}\;=\;\frac{2D^{2}}{Y}$$

Where: D = the longest linear distance of antenna (1.8 m) Y= wavelength (0.33 m).

**Figure 1:**
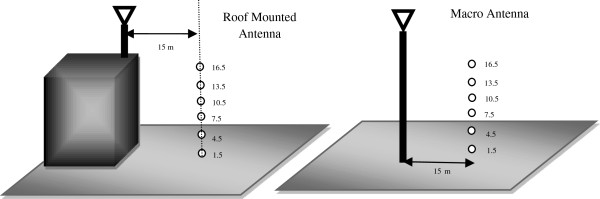
Vertical measurement points around BTS Antennas.

Using the equation above, the effective far field around the antenna starts at 20 m, hence any distances between reactive near field (equal to a wavelength, 0.33 m) and far field (20 m) can be used for accurate measurement in radiating near field. In this case the power density of microwaves in 1.5, 4.5, 7.5, 10.5, 13.5 and 16.5 m height, with 15 m horizontal distance from the center line of antenna were measured (Figure 1). Then the gathered data were analyzed by Excel and SPSS version 19 softwares.

## Results

There are 13 BTS antennae in Hashtgerd city, of which 7 are Irancell operator type and the rest belong to Hamrah-e-Aval. Two types of installations are also studied including Macro antenna and Roof mounted antenna. Of 13 studied antenna, 10 of them were Macro antenna with 3 different heights of (25, 30 and 40 m) and the rest were roof mounted antenna with 3 different overall heights of (20, 25 and 30 m). In addition, all antennae were made by Nokia.

Operator type in its own cannot be a significant determinant of microwave power density around antenna, unless brand type, power consumption and the gain are different. As it was mentioned earlier there are no fundamental differences between different operators antenna. In this case all have the same gain, the same brand and power. Hence the effective factors can be the heights of antenna and traffic load.

**Figure 2:**
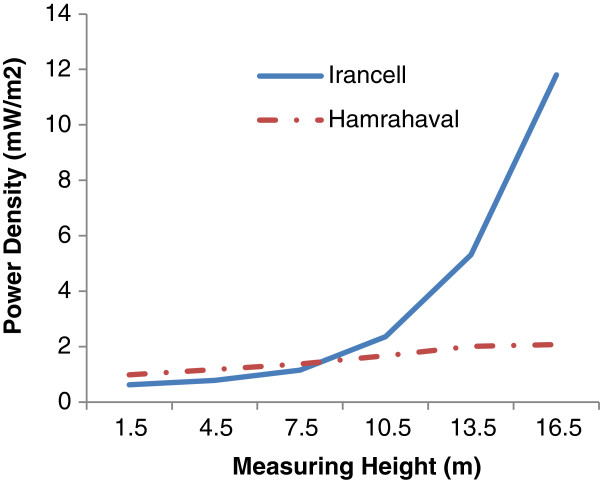
The vertical pattern of microwave radiation around BTS Antenna.

Figure 2 shows the vertical pattern of power density near two different types of operators. Since the data are the average of power density around any different antenna with different installation and heights in each operator’s type, the figure cannot show the operator’s effect on power density produced around antenna. However the figure can qualitatively illustrate the amount of public exposure to microwave radiation produced by different operator in the city.

**Figure 3:**
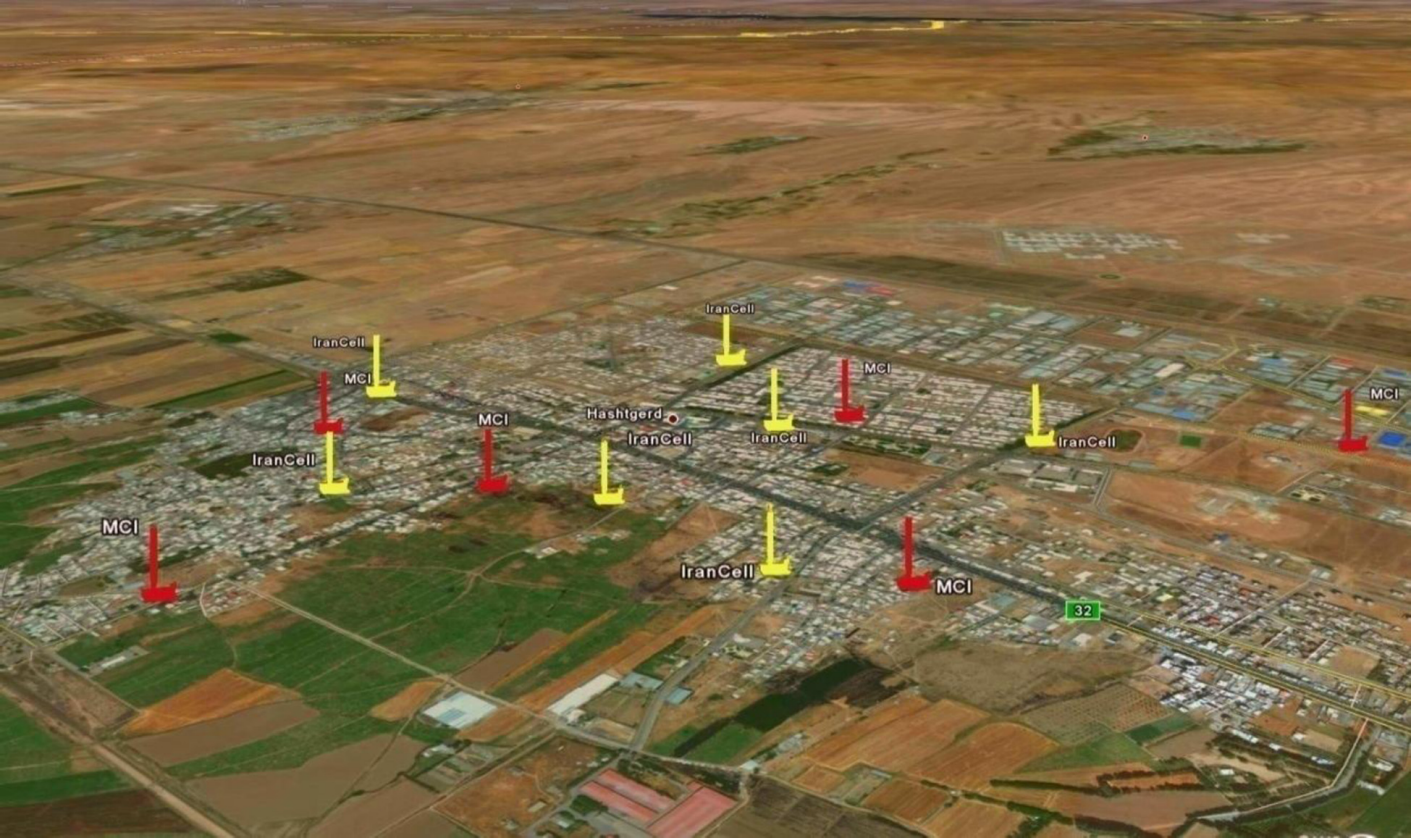
The locations of antenna in Hashtgerd city. Red: Hamrah-e-Aval operator Yellow: Irancell operator.

As one can see in Figure 3, the average of power density around Irancell operator is dramatically higher than that of Hamrah-e-Aval operator at the heights above 8 m. This can be explained by some of the above mentioned variables, something we examine later.

**Figure 4:**
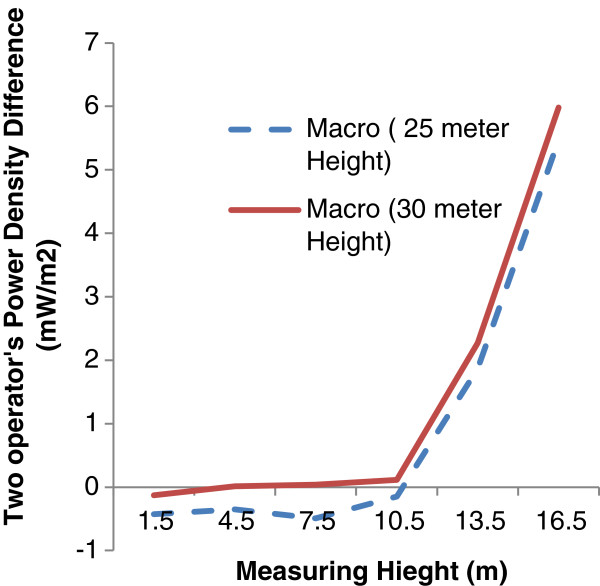
Comparison of difference between power density in different heights near Macro antenna of Irancell and Hamrah-e-Aval operators.

To find the reason behind the dramatic differences of microwave radiation fields around the two studied operators, Figure 4 are introduced. In this part of the study, antenna installation are reduced to just Macro type and the differences between the two operators are examined in antenna with the same heights.

In fact in this figure the effects of antenna installation and antenna height are removed. As it’s clear in the figure the amounts of power density differences at entire different receiver heights is slightly higher in antenna with 30 m heights compared with the shorter antenna. The amount of microwave radiation in terms of power density at the heights above 10 meter is much higher in Irancell operator compared with Hamrah-e-Aval operator. This can be explained by the Irancell installation location and also higher local users. According to the mix model test, a significant statistical relationship was observed between measurement height and the power density in both types of the operators (P = 0.005).

**Figure 5:**
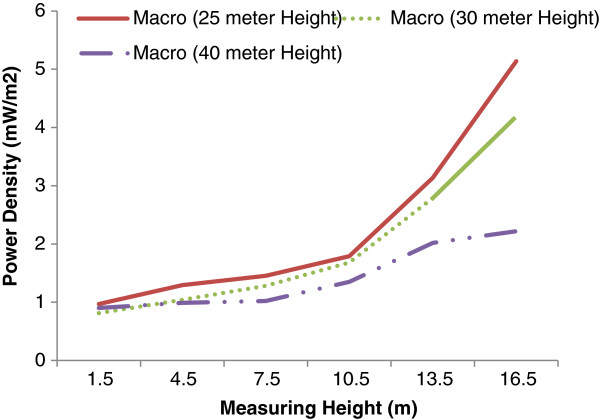
The vertical pattern of microwave radiation around BTS Macro Antenna with different heights.

To investigate the antenna height effect on vertical pattern of microwave power density a separate analysis was considered. Figure 5 shows the results, which is the power density distribution around three different Macro antenna including 25, 30 and 40 m antenna heights.

As it clearly is seen in the figure, the trend of microwave radiation patterns around all three different antennas are almost the same, nevertheless slight differences are seen in higher receiver stations. The amounts of power density in different receiver heights near shorter antenna are significantly higher than that of longer ones. The amounts of wave exposure are increased even further by receiver heights. The explanation of this finding is obvious sine the coordinate for receiver station is ground but the main microwave sources are on the top of the antenna with different heights. Since shortening the antenna provides lesser distances between source and receiver and consequently higher radiation fields will be measured. Therefore reduction of the antenna heights reduced the effective distance between the main source and receiver and as results the amount of power density will be increased. The mix model test indicated a significant statistical relationship between the operator type and the average potential density in different heights. Our findings are consistent with [1,2].

**Figure 6:**
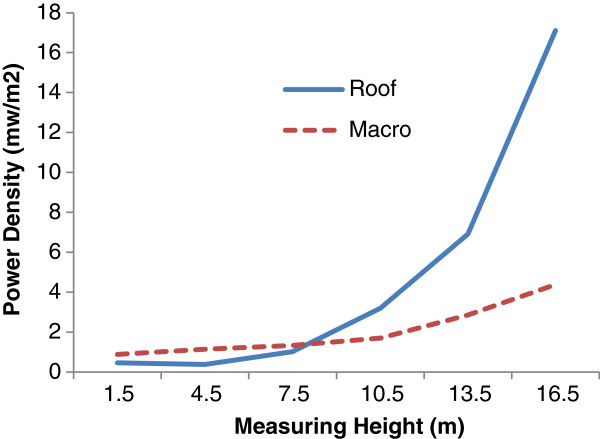
The vertical pattern of microwave radiation around BTS Antenna with different installation.

**Figure 7:**
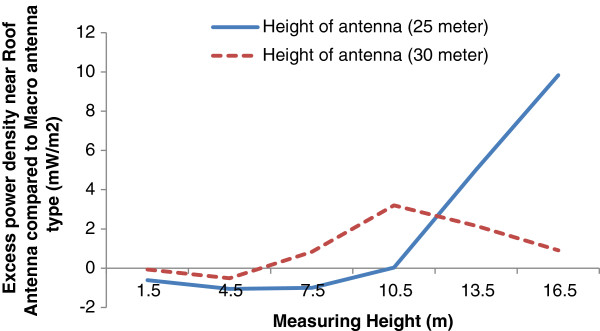
The vertical pattern of excess power density of roof mounted antenna compared with Macro antenna with different heights.

Figures 6 and 7 are illustrated the effect of antenna installation on microwave radiation field around BTS.

The amount of power density at the hieghts above 10 m near Roof mounted antenna much higher than Macro ones according to Figure 6. This can be due to differences between overall heights of the two tested antenna. To investigate this, the above comparison was made for different antenna but with the same overall heights and the results are shown in the Figure 6.

A significant increase in power density of roof mounted antenna with overall height of 25 m is seen at the receiver heights above 10 m while this finding is not true for the antenna with 30 m height. It seems the reason behind this can be explained by the surroundings building wave reflections. In antenna with lower heights, the external building surface provides more reflection and as a results higher electromagnetic field. With increase in height of antenna less reflection and therefore less power increase will be expected some thing is clearly visible in Figure 7.

However, the shadow effect of the buildings provides a condition for roof mounted antenna to have less magnetic field in lower receiver heights.

## Discussion

The lowest amount of measured power density was 0.02 mw/m^2^ belonging to the roof-installed Irancell operator in height of 1.5 m. It seems that the lowest measured amount was the result of buildings interferences so that the structure works as a barrier against transferring wave and obstruct the microwaves pass. Other research about determination of building interferences in potential density indicated density reduction by buildings as a barrier between the antenna and the measuring point, all proving density reduction as the result of the building presence as the interferer [1,11]. Also the highest was related to BTS antennae, Hamrah-e-Aval (First Operator), which was 25 mw/m^2^ at the antenna and it seems that, it was because of the reflection of waves and their increase at the measuring point.

The present study indicated that Irancell antennae were installed in heavily crowded places which had a terrific effect on the exposure to dangerous waves.

On the other hand, operator variables and the vertical distance were incorporated into the regression model. Because the electromagnetic wave propagation had not a normal distribution, it needed to statically be normalized. So the logarithm of the variable was estimated. The below regression equation was the result of analysis where the vertical distance and type of the operator had a significant effect on the logarithm of power density and accounted for 60 per cent of its variations (R2=%60).

(2) PowerDensitymwm2=10X+0.175×(measuringheight)

*X* is a variable to take the type of operator into account, in this case:

$$X\;=\;\left\{\begin{array}{c} 1,\;\mathrm{when}\;\mathrm{operator}\;\mathrm{is}\;\mathrm{Irancell}\\ e^{-0.23-0.112},\;\mathrm{when}\;\mathrm{operator}\;\mathrm{is}\;\mathrm{Hamrah}‒e‒\mathrm{Aval}\; \end{array}\right)$$

This study indicated that Irancell microwave density were higher than that of Hamrah-e-Aval antennae (except for the mentioned densities) (P =0.014).

Also density in both operators rose by increasing the measuring heights (P =0.005).

Because of reporting the maximum density of BTS antennae in as highest and the nearest vertical distance from the antenna, it is recommended that refrain from installing antennae near tall buildings. The study also proved that Irancell antennae needed more care because of the high radiation of microwaves radiated in public.

## Conclusion

The study results indicated that power density increased by decreasing the vertical distance of BTS antennae. Although measured values were under those recommended by the guidelines, taking into account unknown aspect of health effects of this radiation and possibility of chanes in the guidelines, we suggest that building height in proximity of BTS antennae should be considered in getting certifications for antennae installation.

## Competing interests

All authors declare that they have no competing interest.

## Authors’ contributions

MB was the main investigator, designed and performed the study and drafted the manuscript. SN and MRM supervised the study. AM was advisor of the study. SZ helped in English translation and edition. All authors read and approved the final manuscript.
